# Children and adolescents on anti-retroviral therapy in Bulawayo, Zimbabwe: How many are virally suppressed by month six?

**DOI:** 10.12688/f1000research.22744.1

**Published:** 2020-03-16

**Authors:** Silungile Moyo, Ronald Thulani Ncube, Hemant Deepak Shewade, Solwayo Ngwenya, Wedu Ndebele, Kudakwashe Collin Takarinda, Janet Dzangare, Tafadzwa Priscilla Goverwa-Sibanda, Tsitsi Apollo

**Affiliations:** 1 Mpilo Central Hospital, Bulawayo, Zimbabwe; 2 The Union Zimbabwe Office, Harare, Zimbabwe; 3 International Union Against Tuberculosis and Lung Disease (The Union), Paris, France; 4 The Union South East Asia Office, New Delhi, India; 5 AIDS and TB Directorate, Ministry of Health and Child Care, Harare, Zimbabwe; 6 AIDS Health Care Foundation, Harare, Zimbabwe

**Keywords:** Children living with HIV, Adolescent living with HIV, EAC, Mpilo, Operational research, SORT IT

## Abstract

**Background: **Zimbabwe is one of the countries in sub-Saharan Africa disproportionately affected by human immunodeficiency virus. In the “treat all” era, we assessed the gaps in routine viral load (VL) monitoring at six months for children (0-9 years) and adolescents (10-19 years) newly initiated on anti-retroviral therapy (ART) from January 2017 to September 2018 at a large tertiary hospital in Bulawayo.

**Methods: **In this cohort study using secondary data, we considered first VL done within six to nine months of starting therapy as ‘undergoing VL test at six months’. We classified repeat VL≥1000 copies/ml despite enhanced adherence counselling as virally unsuppressed.

**Results:** Of 295 patients initiated on ART, 196 (66%) were children and 99 (34%) adolescents. A total 244 (83%) underwent VL test at six months, with 161 (54%) virally suppressed, 52 (18%) unsuppressed and 82 (28%) with unknown status (due to losses in the cascade). Switch to second line was seen in 35% (18/52). When compared to children, adolescents were less likely to undergo a VL test at six months (73% versus 88%, p=0.002) and more likely to have an unknown VL status (40% versus 22%, p=0.001).

**Conclusion:** At six months of ART, viral suppression was low and losses in the cascade high.

## Introduction

In 2014, the Joint United Nations Programme HIV/AIDS (UNAIDS) announced ambitious new global 90-90-90 fast-track HIV targets for 2020 ^1^. These targets were further supported by the 2016 “treat-all” WHO recommendations ^2^. With the expansion of anti-retroviral treatment (ART) coverage, investments in the global response are shifting towards sustained viral suppression for improved survival and epidemic control. This is in the context of scaling up viral load (VL) monitoring to ensure 90% of people in care are virally suppressed (VL<1000 copies per ml) ^3^. Globally in 2018, only 918 000 (54%) children aged 0–14 years living with HIV received ART ^4^. HIV is among the top 10 leading causes of death among adolescents, a period where sustained adherence is particularly challenging, the only age group where deaths from HIV has not decreased ^5^.

Zimbabwe is disproportionately affected by HIV. In 2017, 1.4 million people were living with HIV, with 5.8% being children 0–14 years ^6^. An estimated 15% on ART have high VL ^7^. The national ART guidelines recommend routine VL monitoring at six and 12 months, and then annually if stable on ART ^8^. The extent to which routine VL monitoring is being implemented specifically for children and adolescents in the “treat-all” era has not been explored in Zimbabwe. We therefore assessed the gaps in routine VL monitoring at six months for children (0–9 years) and adolescents (10–19 years) initiated on ART at a large tertiary hospital in Bulawayo, Zimbabwe.

## Methods

### Study design

We conducted a cohort study involving secondary data.

### Setting

Mpilo Opportunistic Infections (OI) Clinic is within Mpilo Central Hospital in Bulawayo (the second largest city in Zimbabwe). It is a tertiary facility, managing complicated referrals, including patients on second and third line treatment. VL testing is offered as per national guidelines at the hospital HIV laboratory, adjacent to the clinic. Patient data are routinely entered in the electronic point of care database, ART register and patient care booklet ^8^.

First line ART regimen for children <3 years is ABC+3TC+LPV/R or AZT+3TC+LPV/r. Children 3–9 years and adolescents <35kg receive AZT+3TC+NVP or ABC+3TC+EFV, while adolescents >35kg receive TDF+3TC+NVP or EFV ^8^. Those with VL ≥1000 copies/ml at six-months are offered enhanced adherence counseling (EAC), and a second VL test after three months. A repeat VL ≥1000 copies/ml (despite EAC) is classified as virally unsuppressed, and eligible for second line ART ^8^.

### Study population

We included all children (0–9 years) and adolescents (10–19 years) newly initiated on first line ART at Mpilo OI Clinic between January 2017 and September 2018. 

### Data extraction and analysis

We extracted anonymized patient data from electronic patient and laboratory databases, analyzed using STATA (version12.1 STATA Corp., College Station, TX, USA). If data were missing in electronic databases, we referred to paper-based registers and booklets. We defined low CD4 count as follows: CD4 count ≤350 cells/mm ^3^ for children and adolescents >5 years, and CD4% <25% of total lymphocytes for children <5years ^8^. We defined ‘undergoing VL testing at six months’ as those with first VL tests done within six to nine months of starting ART. Comparisons were made between children and adolescents using chi squared test. 

### Ethics

We received ethics approval from Medical Research Council of Zimbabwe (MRCZ E/248), The Union Ethics Advisory Group, Paris, France (EAG 52/19) and the Mpilo Hospital Ethics Board. As the study involved review of secondary data, the ethics committee(s) waived the need for written informed consent.

## Results

Of 295 patients initiated on ART during the study period, 196 (66%) were children and 99 (34%) adolescents. A total 141 (48%) were boys, 209 (71%) were WHO stage I or II, and 119 (40%) had severe anemia. Baseline CD4 count was available for 188, among whom 94 (50%) had low CD4 cell count.

**Figure 1.**
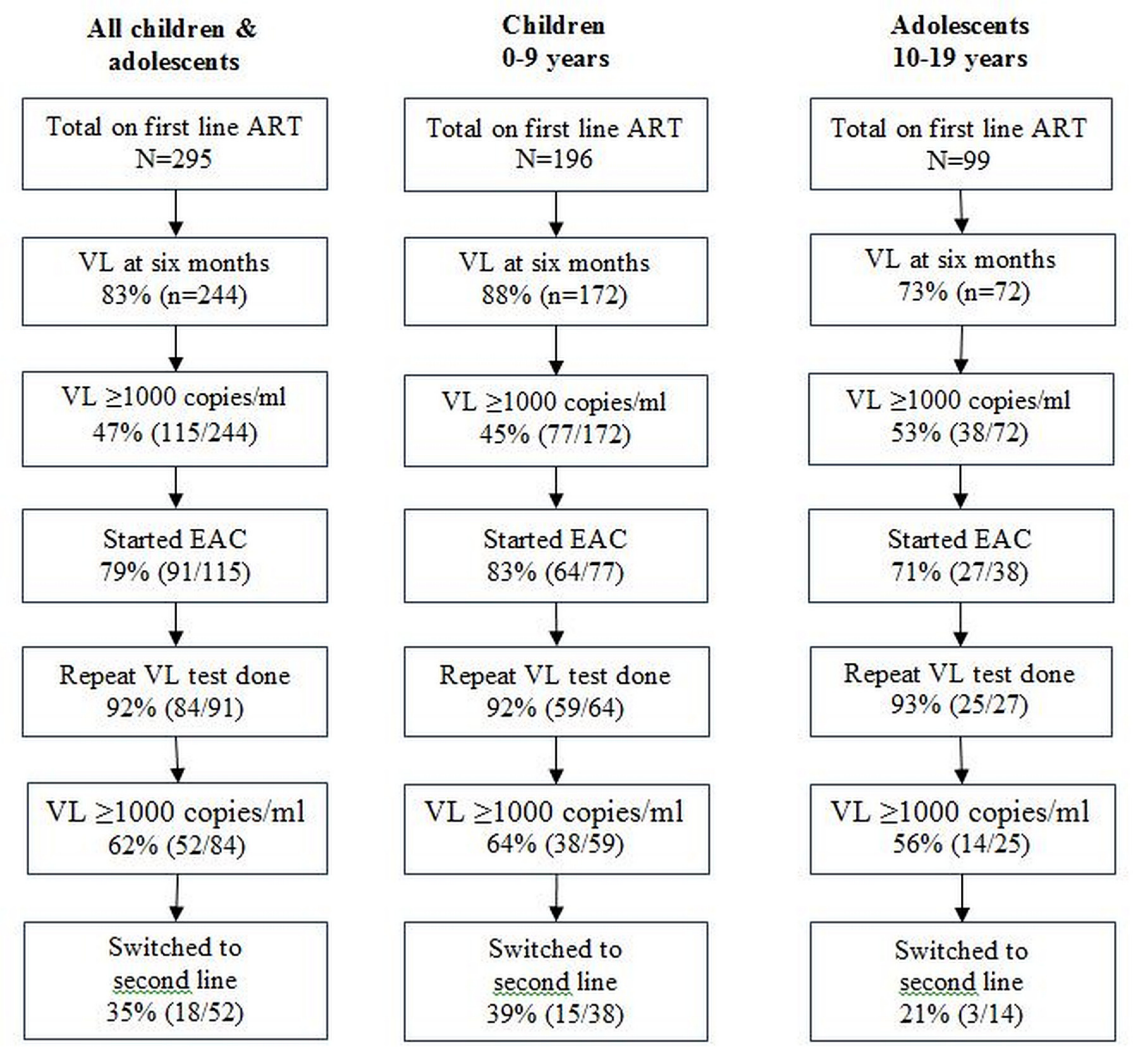
Viral load monitoring cascade for children and adolescents with HIV newly initiated on ART during January 2017 to 30 September 2019 at Mpilo Central Hospital, Bulawayo, Zimbabwe. HIV=human immunodeficiency virus; ART=antiretroviral therapy; VL=viral load; EAC= enhanced adherence counselling.

Of 295, a total of 244 (83%) underwent VL test at six months, which was significantly lower among adolescents when compared to children (73% versus 88%, p=0.002) ( Figure 1). Of 295, 52 (18%) were virally unsuppressed, 161 (54%) virally suppressed and 82 (28%) unknown (unknown due to loss to follow up after ART initiation). Unsuppressed VL was not different among children and adolescents, though unknown VL suppression status, was higher among adolescents (40% versus 22%, p=0.001) ( Table 1). Switch to second line was among 18 out of 52 eligible (35%), with no significant difference between adolescents and children (21% versus 39%, p=0.376) ( Figure 1).

**Table 1.** Viral suppression at six months among children and adolescents with HIV newly initiated on ART during January 2017 to 30 September 2019 at Mpilo Central Hospital, Bulawayo, Zimbabwe.

	Overall (0–19 years)		Children (0–9 years)		Adolescents (10–19 years)	
	**N**	**(%)**	**N**	**(%)**	**N**	**(%)**
Total	295	(100)	196	(100)	99	(100)
Virally unsuppressed *	52	(18)	38	(19)	14	(14)
Virally suppressed **	161	(54)	116	(59)	45	(46)
Unknown ***	82	(28)	42	(22)	40	(40)

Col% HIV=human immunodeficiency virus; ART=antiretroviral therapy. *repeat VL≥1000 copies per ml despite enhanced adherence counseling; **VL<1000 at six months or after repeat VL testing (post enhanced adherence counseling); ***not fitting into any of the above two categories, represents children or adolescents that were lost at any point in the cascade.

## Discussion

This is one of the first studies from Zimbabwe attempting to assess the extent to which routine VL monitoring at six months is being implemented, specifically for children and adolescents in the “treat-all” era. 

Overall, one in five of those initiated on ART were virally unsuppressed at six months. The true estimate could be higher considering viral suppression was unknown for one-third of children. High unsuppressed VL and the observed attrition along the care cascade, undermines the last ‘90’ of the UNAIDS 90-90-90 targets in this special sub-population ^1^. In this study, adolescents were more likely to be lost in the cascade compared to children, calling for focussed interventions for this sub-group. This is inspite of a comprehensive adolescent ART program at the clinic, and suggests a reversal of gains made earlier in the progarm ^9^.

Compared to findings in Harare, where two-thirds of those virally unsuppressed were switched to second line ART, only one-third were switched in our study ^10^. Adherence to national ART guidelines should be an important priority focus in routine clinical mentorship ^11^.

Four in five underwent VL test by six months and EAC in our study, consistent with findings in Harare ^10^. However, we found high proportion with VL≥1000 copies/ml at first and repeat testing. In Swaziland, children and adolescents were more likely to have high VLs and the least likely to achieve viral suppression. This calls for ART treatment support to address adherence problems of children and adolescents ^12^.

The study had some limitations. Missing last visit dates prevented computation of proportion undergoing VL test among those retained at six-months. Missing baseline characteristics precluded analysis of factors associated with not undergoing a VL test and unsuppressed VL.

In conclusion, our study points to gaps in VL monitoring among children and adolescents in Bulawayo. Future studies are needed to understand reasons for attrition along the care cascade to better target interventions.

## Data availability

### Underlying data

Figshare: Dataset for Moyo S *et al.* study: https://doi.org/10.6084/m9.figshare.c.4884726.v1 ^13^

File ‘silungiledata’ contains all de-identified variables extracted for this study, alongside a codebook explaining all fields and field values.

Data are available under the terms of the Creative Commons Zero "No rights reserved" data waiver (CC0 1.0 Public domain dedication).
