# Supplementary material for: Children and adolescents on anti-retroviral therapy in Bulawayo, Zimbabwe: How many are virally suppressed by month six?
Source: F1000Res. 2020 Mar 16;9:191. [Version 1] doi: 10.12688/f1000research.22744.1 (PMC7194453; doi:10.12688/f1000research.22744.1)
Supplement: Peer review history

# Peer review: Reviewer response for version 1

**DOI:** 10.5256/f1000research.25113.r61603

**Published:** 2020-05-26

**Authors:** Catherine Kegakilwe Koofhethile

**Affiliations:** 1 Botswana Harvard AIDS Institute Partnership, Gaborone, Botswana; 2 Harvard T.H. Chan School of Public Health, Boston, MA, USA

I think that this manuscript is addressing a very important gap in knowledge that is relevant for the current ‘treat all’ recommendations.

 They accessed the gaps in routine viral load monitoring at six months for children and adolescents who initiated antiretroviral therapy in a hospital in Zimbabwe. Their sample number is good enough for this analysis. The manuscript is very well written, it is very clear and concise. The study was based on analysis of secondary data which was approved by IRB.

 I only have one comment that need clarification- the authors keep comparing their analysis with a study done in Harare and it is not clear whether this study that they are comparing to was conducted on adult population or the same population as they describe in their analysis. This needs to be clarified. In addition, they need to explain what could be accounting for the differences found.

Is the work clearly and accurately presented and does it cite the current literature?

Yes

If applicable, is the statistical analysis and its interpretation appropriate?

Yes

Are all the source data underlying the results available to ensure full reproducibility?

No source data required

Is the study design appropriate and is the work technically sound?

Yes

Are the conclusions drawn adequately supported by the results?

Yes

Are sufficient details of methods and analysis provided to allow replication by others?

Yes

Reviewer Expertise:

Immunology, HIV,

I confirm that I have read this submission and believe that I have an appropriate level of expertise to confirm that it is of an acceptable scientific standard, however I have significant reservations, as outlined above.

# Peer review: Reviewer response for version 1

**DOI:** 10.5256/f1000research.25113.r61601

**Published:** 2020-04-28

**Authors:** Brian van Wyk

**Affiliations:** 1 School of Public Health, University of the Western Cape, Cape Town, South Africa

The study reports on viral load monitoring at 6 months for of children and adolescents who were initiated on HIV treatment in a tertiary hospital in Bulawayo. The study is important because of the HIV epidemic in Zimbabwe, and the need to reach the third 90 of UNAIDS 90-90-90 targets.

 The methodology is sound and clearly reported on. Appropriate statistical analysis is done, and these are aligned with the objectives of the study.

 Few other sociodemographic and clinical factors were collected and analysed; which is a limitation to the study. This should be indicated. 

 In the discussion, enhanced adherence counseling is mentioned as being implemented in the hospital. However, little information on this is provided in the background. Also, it would be useful if the analysis could report on how many of the current cohort received enhanced adherence counseling.

Is the work clearly and accurately presented and does it cite the current literature?

Yes

If applicable, is the statistical analysis and its interpretation appropriate?

Yes

Are all the source data underlying the results available to ensure full reproducibility?

Yes

Is the study design appropriate and is the work technically sound?

Yes

Are the conclusions drawn adequately supported by the results?

Yes

Are sufficient details of methods and analysis provided to allow replication by others?

Yes

Reviewer Expertise:

HIV/AIDS; Health Systems;  Adolescent Health

I confirm that I have read this submission and believe that I have an appropriate level of expertise to confirm that it is of an acceptable scientific standard.

# Response

**Published:** 2020-04-29

**Authors:** Hemant Deepak Shewade

**Affiliations:** International Union Against Tuberculosis and Lung Disease, India

Thank you very much for your constructive comments. 

Reviewer

Few other sociodemographic and clinical factors were collected and analysed; which is a limitation to the study. 

Authors

We have included this and mentioned it as a limitation in the last but one paragraph of the discussion section. We hope this is fine. 

Reviewer

In the discussion, enhanced adherence counseling is mentioned as being implemented in the hospital. However, little information on this is provided in the background. Also, it would be useful if the analysis could report on how many of the current cohort received enhanced adherence counseling.

Authors

We have information regarding when enhaned adherence conselling is provided in the settings section. We hope this is fine. We have provided information regarding how many recevied enhanced adherence counselling in this cohort. Please see figure 1.

We hope our response is satisfactory and the current version does not require any edits. Hence, we are not submitting a revised manuscript.

# Peer review: Reviewer response for version 1

**DOI:** 10.5256/f1000research.25113.r61604

**Published:** 2020-04-08

**Authors:** Obinna Ikechukwu Ekwunife

**Affiliations:** 1 Department of Clinical Pharmacy and Pharmacy Management, Nnamdi Azikiwe University, Awka, Nigeria

This short report aimed to assess the gaps in routine VL monitoring at six months for children (0-9 years) and adolescents (10-19 years) newly initiated on anti-retroviral therapy from Jan 2017 to Sep 2018.

 This study is essential as such data is needed to assess how programs are fairing with regards to the UNAIDS 90-90-90 target.

 The study was succinctly reported. All the essentials results based on their study objective were addressed. The study should be accepted.

Is the work clearly and accurately presented and does it cite the current literature?

Yes

If applicable, is the statistical analysis and its interpretation appropriate?

Yes

Are all the source data underlying the results available to ensure full reproducibility?

Yes

Is the study design appropriate and is the work technically sound?

Yes

Are the conclusions drawn adequately supported by the results?

Yes

Are sufficient details of methods and analysis provided to allow replication by others?

Yes

Reviewer Expertise:

Pharmacoeconomics, evidence-based public health

I confirm that I have read this submission and believe that I have an appropriate level of expertise to confirm that it is of an acceptable scientific standard.

# Response

**Published:** 2020-04-29

**Authors:** Hemant Deepak Shewade

**Affiliations:** International Union Against Tuberculosis and Lung Disease, India

Thank you for the constructive comments
